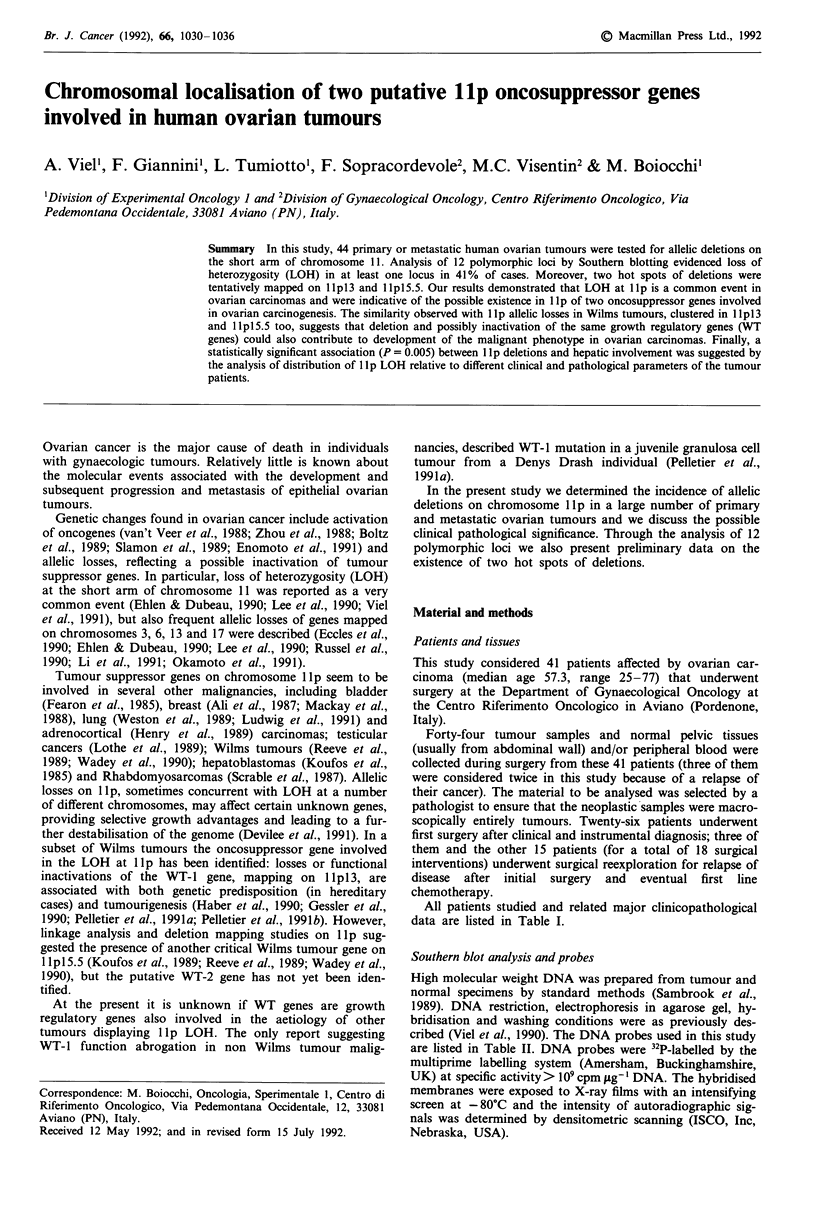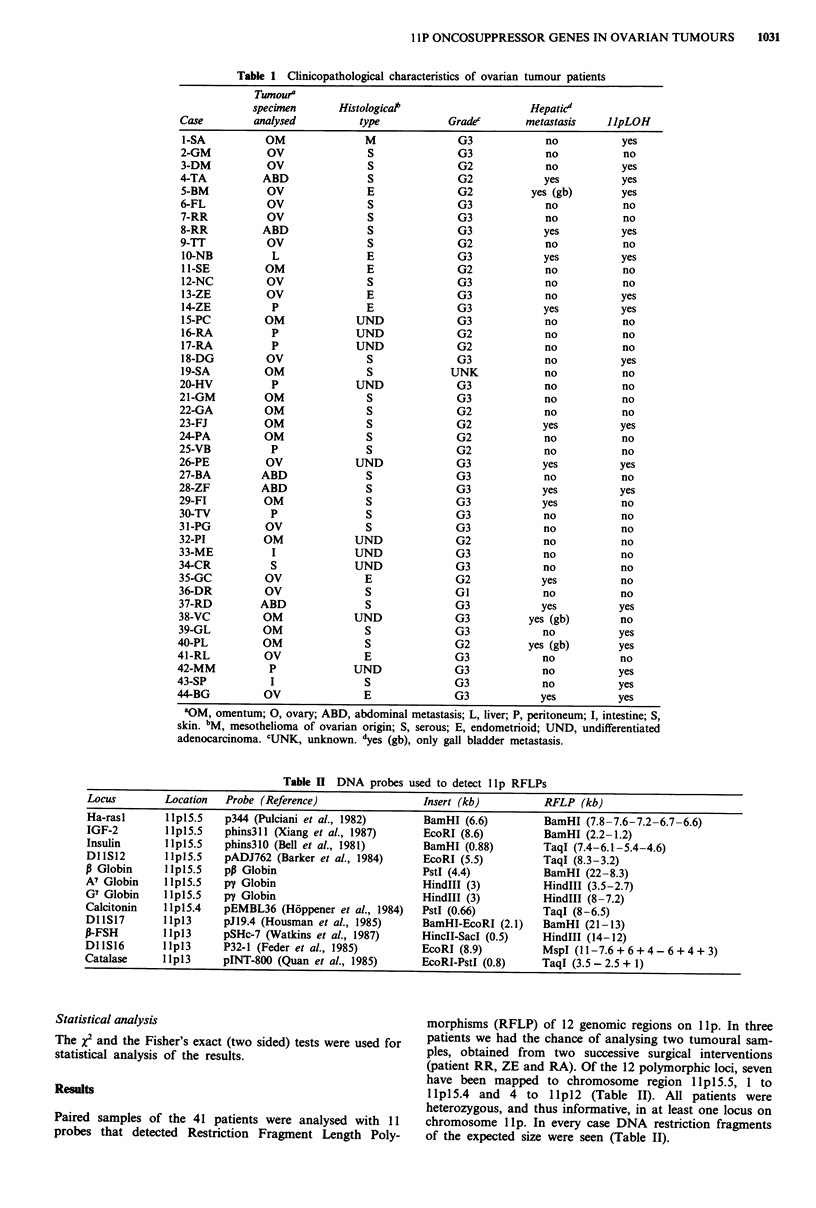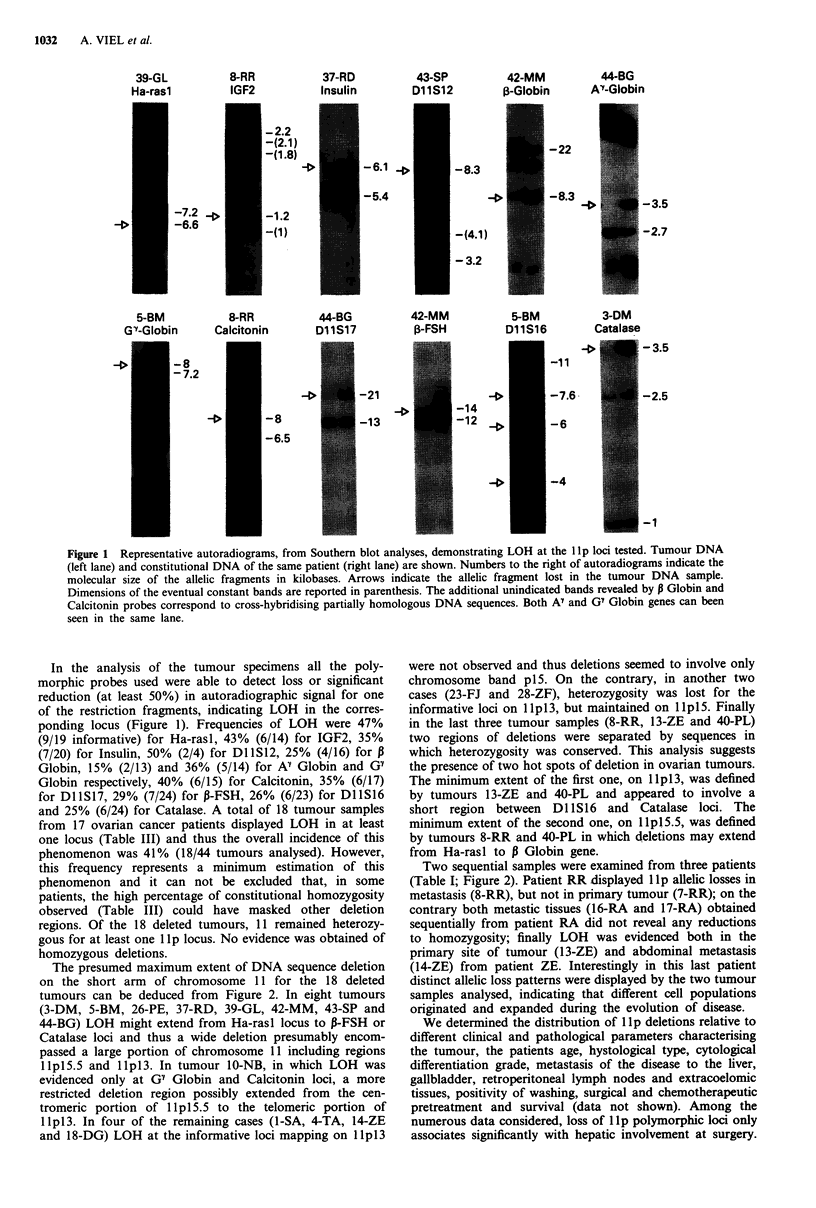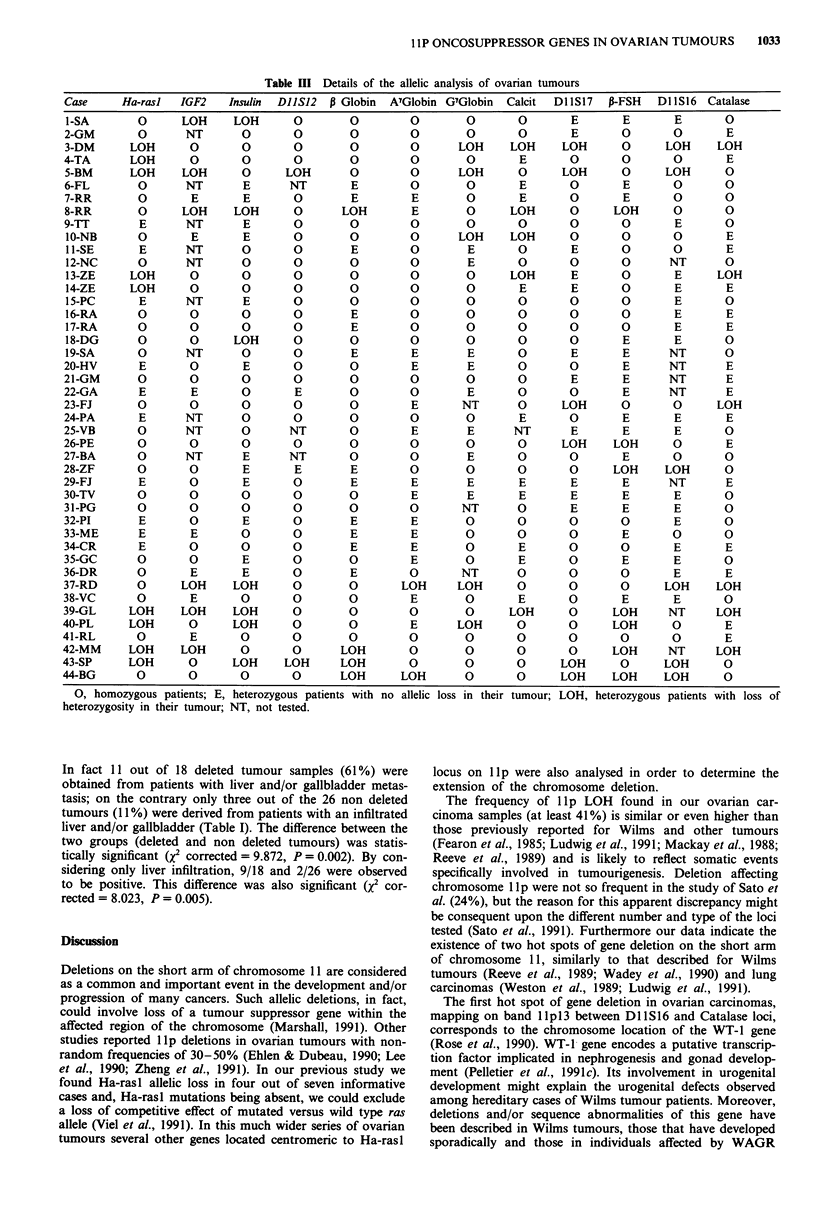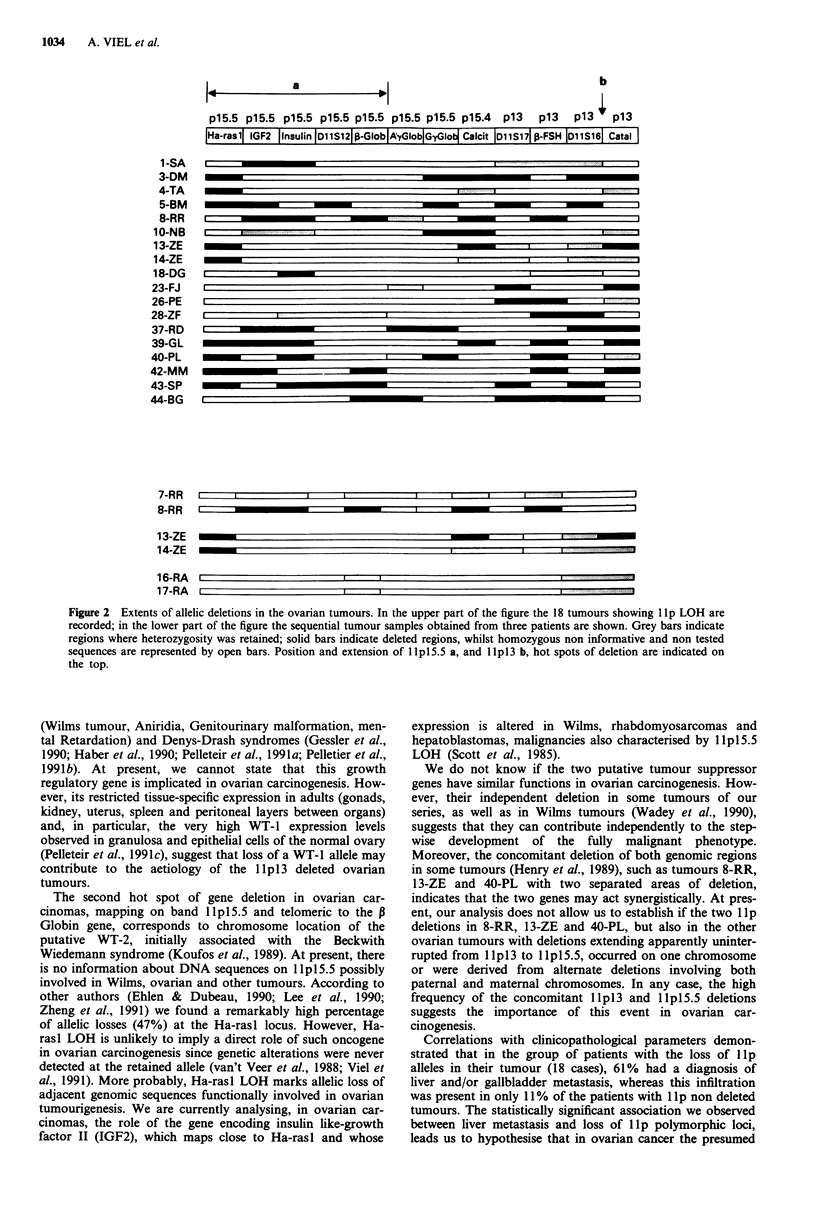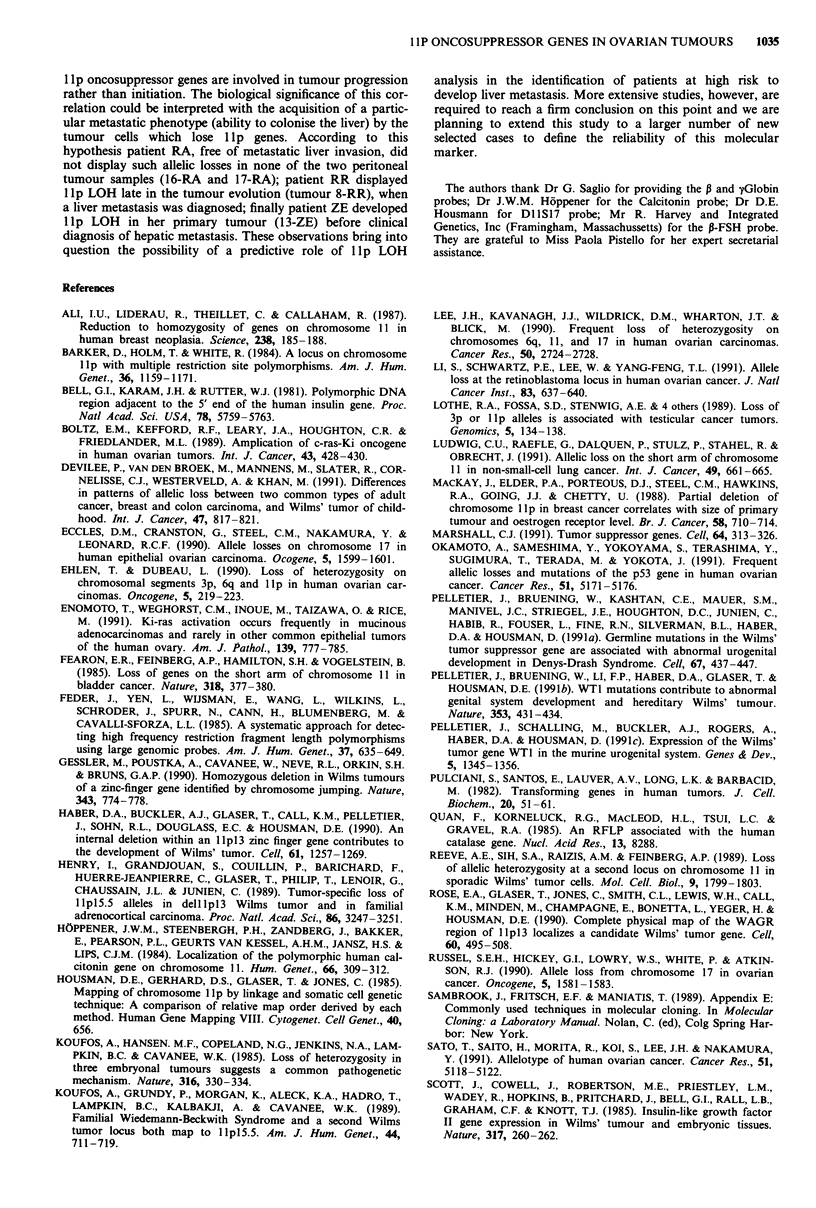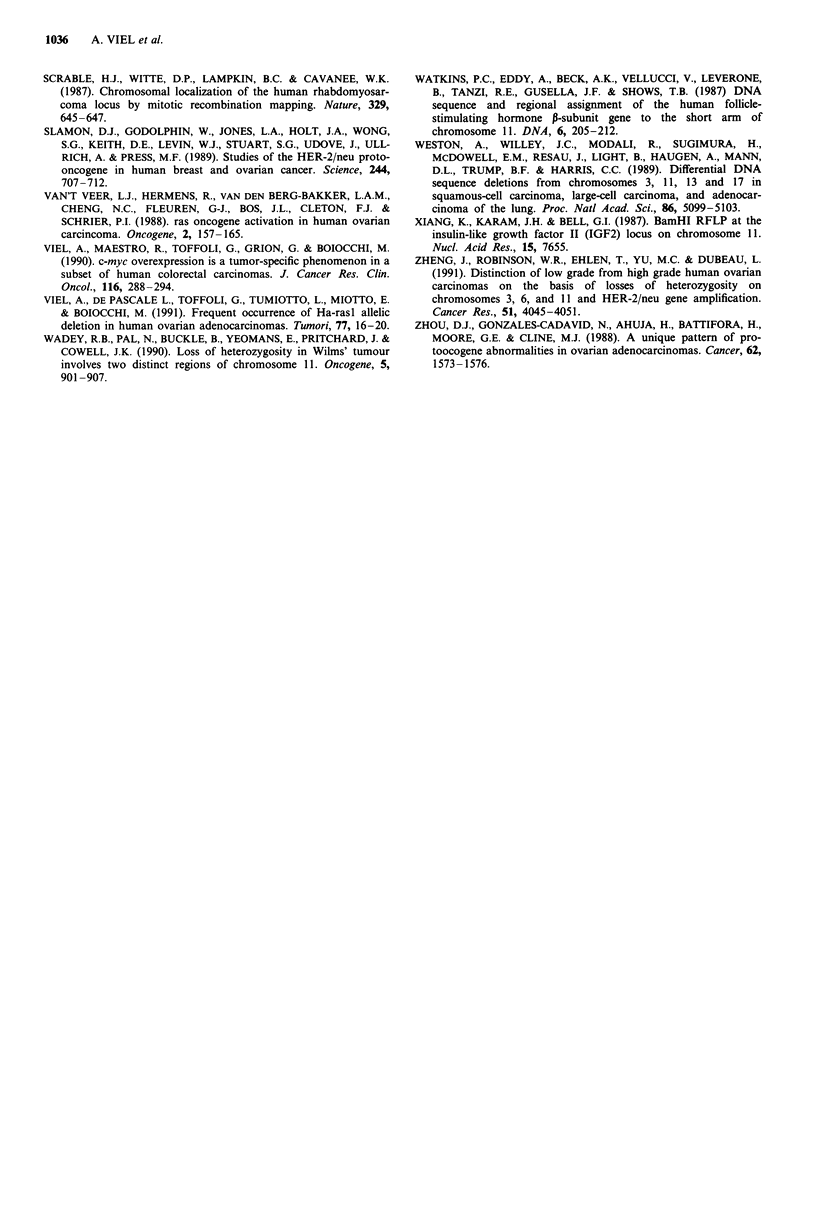# Chromosomal localisation of two putative 11p oncosuppressor genes involved in human ovarian tumours.

**DOI:** 10.1038/bjc.1992.405

**Published:** 1992-12

**Authors:** A. Viel, F. Giannini, L. Tumiotto, F. Sopracordevole, M. C. Visentin, M. Boiocchi

**Affiliations:** Division of Experimental Oncology 1, Centro Riferimento Oncologico, Aviano (PN), Italy.

## Abstract

**Images:**


Br. J. Cancer (1992), 66, 1030-1036 © Macmillan Press Ltd., 1992

### Chromosomal localisation of two putative 11p oncosuppressor genes involved in human ovarian tumours

A. Viel[1], F. Giannini[1], L. Tumiotto[1], F. Sopracordevole[2], M.C. Visentin[2] & M. Boiocchi[1]

[1]*Division of Experimental Oncology 1 and* [2]*Division of Gynaecological Oncology, Centro Riferimento Oncologico, Via Pedemontana Occidentale, 33081 Aviano (PN), Italy.*

**Summary** In this study, 44 primary or metastatic human ovarian tumours were tested for allelic deletions on the short arm of chromosome 11. Analysis of 12 polymorphic loci by Southern blotting evidenced loss of heterozygosity (LOH) in at least one locus in 41% of cases. Moreover, two hot spots of deletions were tentatively mapped on 11p13 and 11p15.5. Our results demonstrated that LOH at 11p is a common event in ovarian carcinomas and were indicative of the possible existence in 11p of two oncosuppressor genes involved in ovarian carcinogenesis. The similarity observed with 11p allelic losses in Wilms tumours, clustered in 11p13 and 11p15.5 too, suggests that deletion and possibly inactivation of the same growth regulatory genes (WT genes) could also contribute to development of the malignant phenotype in ovarian carcinomas. Finally, a statistically significant association ($P = 0.005$) between 11p deletions and hepatic involvement was suggested by the analysis of distribution of 11p LOH relative to different clinical and pathological parameters of the tumour patients.

Ovarian cancer is the major cause of death in individuals with gynaecologic tumours. Relatively little is known about the molecular events associated with the development and subsequent progression and metastasis of epithelial ovarian tumours.

Genetic changes found in ovarian cancer include activation of oncogenes (van't Veer *et al.*, 1988; Zhou *et al.*, 1988; Boltz *et al.*, 1989; Slamon *et al.*, 1989; Enomoto *et al.*, 1991) and allelic losses, reflecting a possible inactivation of tumour suppressor genes. In particular, loss of heterozygosity (LOH) at the short arm of chromosome 11 was reported as a very common event (Ehlen & Dubeau, 1990; Lee *et al.*, 1990; Viel *et al.*, 1991), but also frequent allelic losses of genes mapped on chromosomes 3, 6, 13 and 17 were described (Eccles *et al.*, 1990; Ehlen & Dubeau, 1990; Lee *et al.*, 1990; Russel *et al.*, 1990; Li *et al.*, 1991; Okamoto *et al.*, 1991).

Tumour suppressor genes on chromosome 11p seem to be involved in several other malignancies, including bladder (Fearon *et al.*, 1985), breast (Ali *et al.*, 1987; Mackay *et al.*, 1988), lung (Weston *et al.*, 1989; Ludwig *et al.*, 1991) and adrenocortical (Henry *et al.*, 1989) carcinomas; testicular cancers (Lothe *et al.*, 1989); Wilms tumours (Reeve *et al.*, 1989; Wadey *et al.*, 1990); hepatoblastomas (Koufos *et al.*, 1985) and Rhabdomyosarcomas (Scrable *et al.*, 1987). Allelic losses on 11p, sometimes concurrent with LOH at a number of different chromosomes, may affect certain unknown genes, providing selective growth advantages and leading to a further destabilisation of the genome (Devilee *et al.*, 1991). In a subset of Wilms tumours the oncosuppressor gene involved in the LOH at 11p has been identified: losses or functional inactivations of the WT-1 gene, mapping on 11p13, are associated with both genetic predisposition (in hereditary cases) and tumourigenesis (Haber *et al.*, 1990; Gessler *et al.*, 1990; Pelletier *et al.*, 1991*a*; Pelletier *et al.*, 1991*b*). However, linkage analysis and deletion mapping studies on 11p suggested the presence of another critical Wilms tumour gene on 11p15.5 (Koufos *et al.*, 1989; Reeve *et al.*, 1989; Wadey *et al.*, 1990), but the putative WT-2 gene has not yet been identified.

At the present it is unknown if WT genes are growth regulatory genes also involved in the aetiology of other tumours displaying 11p LOH. The only report suggesting WT-1 function abrogation in non Wilms tumour malignancies, described WT-1 mutation in a juvenile granulosa cell tumour from a Denys Drash individual (Pelletier *et al.*, 1991*a*).

In the present study we determined the incidence of allelic deletions on chromosome 11p in a large number of primary and metastatic ovarian tumours and we discuss the possible clinical pathological significance. Through the analysis of 12 polymorphic loci we also present preliminary data on the existence of two hot spots of deletions.

### Material and methods

### *Patients and tissues*

This study considered 41 patients affected by ovarian carcinoma (median age 57.3, range 25-77) that underwent surgery at the Department of Gynaecological Oncology at the Centro Riferimento Oncologico in Aviano (Pordenone, Italy).

Forty-four tumour samples and normal pelvic tissues (usually from abdominal wall) and/or peripheral blood were collected during surgery from these 41 patients (three of them were considered twice in this study because of a relapse of their cancer). The material to be analysed was selected by a pathologist to ensure that the neoplastic samples were macroscopically entirely tumours. Twenty-six patients underwent first surgery after clinical and instrumental diagnosis; three of them and the other 15 patients (for a total of 18 surgical interventions) underwent surgical reexploration for relapse of disease after initial surgery and eventual first line chemotherapy.

All patients studied and related major clinicopathological data are listed in Table I.

### *Southern blot analysis and probes*

High molecular weight DNA was prepared from tumour and normal specimens by standard methods (Sambrook *et al.*, 1989). DNA restriction, electrophoresis in agarose gel, hybridisation and washing conditions were as previously described (Viel *et al.*, 1990). The DNA probes used in this study are listed in Table II. DNA probes were $^{32}P$-labelled by the multiprime labelling system (Amersham, Buckinghamshire, UK) at specific activity $> 10^9$ cpm $\mu g^{-1}$ DNA. The hybridised membranes were exposed to X-ray films with an intensifying screen at $-80°C$ and the intensity of autoradiographic signals was determined by densitometric scanning (ISCO, Inc, Nebraska, USA).

Correspondence: M. Boiocchi, Oncologia, Sperimentale 1, Centro di Riferimento Oncologico, Via Pedemontana Occidentale, 12, 33081 Aviano (PN), Italy.
Received 12 May 1992; and in revised form 15 July 1992.

**Table I** Clinicopathological characteristics of ovarian tumour patients

| *Case* | *Tumour[a] specimen analysed* | *Histological[b] type* | *Grade[c]* | *Hepatic[d] metastasis* | *11pLOH* |
|---|---|---|---|---|---|
| 1-SA | OM | M | G3 | no | yes |
| 2-GM | OV | S | G3 | no | no |
| 3-DM | OV | S | G2 | no | yes |
| 4-TA | ABD | S | G2 | yes | yes |
| 5-BM | OV | E | G2 | yes (gb) | yes |
| 6-FL | OV | S | G3 | no | no |
| 7-RR | OV | S | G3 | no | no |
| 8-RR | ABD | S | G3 | yes | yes |
| 9-TT | OV | S | G2 | no | no |
| 10-NB | L | E | G3 | yes | yes |
| 11-SE | OM | E | G2 | no | no |
| 12-NC | OV | S | G3 | no | no |
| 13-ZE | OV | E | G3 | no | yes |
| 14-ZE | P | E | G3 | yes | yes |
| 15-PC | OM | UND | G3 | no | no |
| 16-RA | P | UND | G2 | no | no |
| 17-RA | P | UND | G2 | no | no |
| 18-DG | OV | S | G3 | no | yes |
| 19-SA | OM | S | UNK | no | no |
| 20-HV | P | UND | G3 | no | no |
| 21-GM | OM | S | G3 | no | no |
| 22-GA | OM | S | G2 | no | no |
| 23-FJ | OM | S | G2 | yes | yes |
| 24-PA | OM | S | G2 | no | no |
| 25-VB | P | S | G2 | no | no |
| 26-PE | OV | UND | G3 | yes | yes |
| 27-BA | ABD | S | G3 | no | no |
| 28-ZF | ABD | S | G3 | yes | yes |
| 29-FI | OM | S | G3 | yes | no |
| 30-TV | P | S | G3 | no | no |
| 31-PG | OV | S | G3 | no | no |
| 32-PI | OM | UND | G2 | no | no |
| 33-ME | I | UND | G3 | no | no |
| 34-CR | S | UND | G3 | no | no |
| 35-GC | OV | E | G2 | yes | no |
| 36-DR | OV | S | G1 | no | no |
| 37-RD | ABD | S | G3 | yes | yes |
| 38-VC | OM | UND | G3 | yes (gb) | no |
| 39-GL | OM | S | G3 | no | yes |
| 40-PL | OM | S | G2 | yes (gb) | yes |
| 41-RL | OV | E | G3 | no | no |
| 42-MM | P | UND | G3 | no | yes |
| 43-SP | I | S | G3 | no | yes |
| 44-BG | OV | E | G3 | yes | yes |

[a]OM, omentum; O, ovary; ABD, abdominal metastasis; L, liver; P, peritoneum; I, intestine; S, skin. [b]M, mesothelioma of ovarian origin; S, serous; E, endometrioid; UND, undifferentiated adenocarcinoma. [c]UNK, unknown. [d]yes (gb), only gall bladder metastasis.

**Table II** DNA probes used to detect 11p RFLPs

| *Locus* | *Location* | *Probe (Reference)* | *Insert (kb)* | *RFLP (kb)* |
|---|---|---|---|---|
| Ha-ras1 | 11p15.5 | p344 (Pulciani *et al.*, 1982) | BamHI (6.6) | BamHI (7.8-7.6-7.2-6.7-6.6) |
| IGF-2 | 11p15.5 | phins311 (Xiang *et al.*, 1987) | EcoRI (8.6) | BamHI (2.2-1.2) |
| Insulin | 11p15.5 | phins310 (Bell *et al.*, 1981) | BamHI (0.88) | TaqI (7.4-6.1-5.4-4.6) |
| D11S12 | 11p15.5 | pADJ762 (Barker *et al.*, 1984) | EcoRI (5.5) | TaqI (8.3-3.2) |
| β Globin | 11p15.5 | pβ Globin | PstI (4.4) | BamHI (22-8.3) |
| A$^{\gamma}$ Globin | 11p15.5 | pγ Globin | HindIII (3) | HindIII (3.5-2.7) |
| G$^{\gamma}$ Globin | 11p15.5 | pγ Globin | HindIII (3) | HindIII (8-7.2) |
| Calcitonin | 11p15.4 | pEMBL36 (Höppener *et al.*, 1984) | PstI (0.66) | TaqI (8-6.5) |
| D11S17 | 11p13 | pJ19.4 (Housman *et al.*, 1985) | BamHI-EcoRI (2.1) | BamHI (21-13) |
| β-FSH | 11p13 | pSHc-7 (Watkins *et al.*, 1987) | HincII-SacI (0.5) | HindIII (14-12) |
| D11S16 | 11p13 | P32-1 (Feder *et al.*, 1985) | EcoRI (8.9) | MspI (11-7.6 + 6 + 4 - 6 + 4 + 3) |
| Catalase | 11p13 | pINT-800 (Quan *et al.*, 1985) | EcoRI-PstI (0.8) | TaqI (3.5 - 2.5 + 1) |

### *Statistical analysis*

The $\chi^2$ and the Fisher's exact (two sided) tests were used for statistical analysis of the results.

### Results

Paired samples of the 41 patients were analysed with 11 probes that detected Restriction Fragment Length Polymorphisms (RFLP) of 12 genomic regions on 11p. In three patients we had the chance of analysing two tumoural samples, obtained from two successive surgical interventions (patient RR, ZE and RA). Of the 12 polymorphic loci, seven have been mapped to chromosome region 11p15.5, 1 to 11p15.4 and 4 to 11p12 (Table II). All patients were heterozygous, and thus informative, in at least one locus on chromosome 11p. In every case DNA restriction fragments of the expected size were seen (Table II).

**Figure 1** Representative autoradiograms, from Southern blot analyses, demonstrating LOH at the 11p loci tested. Tumour DNA (left lane) and constitutional DNA of the same patient (right lane) are shown. Numbers to the right of autoradiograms indicate the molecular size of the allelic fragments in kilobases. Arrows indicate the allelic fragment lost in the tumour DNA sample. Dimensions of the eventual constant bands are reported in parenthesis. The additional unindicated bands revealed by β Globin and Calcitonin probes correspond to cross-hybridising partially homologous DNA sequences. Both $A^{\gamma}$ and $G^{\gamma}$ Globin genes can been seen in the same lane.

In the analysis of the tumour specimens all the polymorphic probes used were able to detect loss or significant reduction (at least 50%) in autoradiographic signal for one of the restriction fragments, indicating LOH in the corresponding locus (Figure 1). Frequencies of LOH were 47% (9/19 informative) for Ha-rasl, 43% (6/14) for IGF2, 35% (7/20) for Insulin, 50% (2/4) for D11S12, 25% (4/16) for β Globin, 15% (2/13) and 36% (5/14) for $A^{\gamma}$ Globin and $G^{\gamma}$ Globin respectively, 40% (6/15) for Calcitonin, 35% (6/17) for D11S17, 29% (7/24) for β-FSH, 26% (6/23) for D11S16 and 25% (6/24) for Catalase. A total of 18 tumour samples from 17 ovarian cancer patients displayed LOH in at least one locus (Table III) and thus the overall incidence of this phenomenon was 41% (18/44 tumours analysed). However, this frequency represents a minimum estimation of this phenomenon and it can not be excluded that, in some patients, the high percentage of constitutional homozygosity observed (Table III) could have masked other deletion regions. Of the 18 deleted tumours, 11 remained heterozygous for at least one 11p locus. No evidence was obtained of homozygous deletions.

The presumed maximum extent of DNA sequence deletion on the short arm of chromosome 11 for the 18 deleted tumours can be deduced from Figure 2. In eight tumours (3-DM, 5-BM, 26-PE, 37-RD, 39-GL, 42-MM, 43-SP and 44-BG) LOH might extend from Ha-rasl locus to β-FSH or Catalase loci and thus a wide deletion presumably encompassed a large portion of chromosome 11 including regions 11p15.5 and 11p13. In tumour 10-NB, in which LOH was evidenced only at $G^{\gamma}$ Globin and Calcitonin loci, a more restricted deletion region possibly extended from the centromeric portion of 11p15.5 to the telomeric portion of 11p13. In four of the remaining cases (1-SA, 4-TA, 14-ZE and 18-DG) LOH at the informative loci mapping on 11p13 were not observed and thus deletions seemed to involve only chromosome band p15. On the contrary, in another two cases (23-FJ and 28-ZF), heterozygosity was lost for the informative loci on 11p13, but maintained on 11p15. Finally in the last three tumour samples (8-RR, 13-ZE and 40-PL) two regions of deletions were separated by sequences in which heterozygosity was conserved. This analysis suggests the presence of two hot spots of deletion in ovarian tumours. The minimum extent of the first one, on 11p13, was defined by tumours 13-ZE and 40-PL and appeared to involve a short region between D11S16 and Catalase loci. The minimum extent of the second one, on 11p15.5, was defined by tumours 8-RR and 40-PL in which deletions may extend from Ha-rasl to β Globin gene.

Two sequential samples were examined from three patients (Table I; Figure 2). Patient RR displayed 11p allelic losses in metastasis (8-RR), but not in primary tumour (7-RR); on the contrary both metastic tissues (16-RA and 17-RA) obtained sequentially from patient RA did not reveal any reductions to homozygosity; finally LOH was evidenced both in the primary site of tumour (13-ZE) and abdominal metastasis (14-ZE) from patient ZE. Interestingly in this last patient distinct allelic loss patterns were displayed by the two tumour samples analysed, indicating that different cell populations originated and expanded during the evolution of disease.

We determined the distribution of 11p deletions relative to different clinical and pathological parameters characterising the tumour, the patients age, hystological type, cytological differentiation grade, metastasis of the disease to the liver, gallbladder, retroperitoneal lymph nodes and extracoelomic tissues, positivity of washing, surgical and chemotherapeutic pretreatment and survival (data not shown). Among the numerous data considered, loss of 11p polymorphic loci only associates significantly with hepatic involvement at surgery.

**Table III** Details of the allelic analysis of ovarian tumours

| *Case* | *Ha-ras1* | *IGF2* | *Insulin* | *D11S12* | β Globin | A'Globin | G'Globin | Calcit | D11S17 | β-FSH | D11S16 | Catalase |
|---|---|---|---|---|---|---|---|---|---|---|---|---|
| 1-SA | O | LOH | LOH | O | O | O | O | O | E | E | E | O |
| 2-GM | O | NT | O | O | O | O | O | O | E | O | O | E |
| 3-DM | LOH | O | O | O | O | O | LOH | LOH | LOH | O | LOH | LOH |
| 4-TA | LOH | O | O | O | O | O | O | E | O | O | O | E |
| 5-BM | LOH | LOH | O | LOH | O | O | LOH | O | LOH | O | LOH | O |
| 6-FL | O | NT | E | NT | E | O | O | E | O | E | O | O |
| 7-RR | O | E | E | O | E | E | O | E | O | E | O | O |
| 8-RR | O | LOH | LOH | O | LOH | E | O | LOH | O | LOH | O | O |
| 9-TT | E | NT | E | O | O | O | O | O | O | O | E | O |
| 10-NB | O | E | E | O | O | O | LOH | LOH | O | O | O | E |
| 11-SE | E | NT | O | O | E | O | E | O | E | O | O | E |
| 12-NC | O | NT | O | O | O | O | E | O | O | O | NT | O |
| 13-ZE | LOH | O | O | O | O | O | O | LOH | E | O | E | LOH |
| 14-ZE | LOH | O | O | O | O | O | O | E | E | O | E | E |
| 15-PC | E | NT | E | O | O | O | O | O | O | O | E | O |
| 16-RA | O | O | O | O | E | O | O | O | O | O | E | E |
| 17-RA | O | O | O | O | E | O | O | O | O | O | E | E |
| 18-DG | O | O | LOH | O | O | O | O | O | O | E | E | O |
| 19-SA | O | NT | O | O | E | E | E | O | E | E | NT | O |
| 20-HV | E | O | E | O | O | E | E | O | O | E | NT | E |
| 21-GM | O | O | O | O | O | O | O | O | E | E | NT | E |
| 22-GA | E | E | O | E | O | O | E | O | O | E | NT | E |
| 23-FJ | O | O | O | O | O | E | NT | O | LOH | O | O | LOH |
| 24-PA | E | NT | O | O | O | O | O | E | O | E | E | E |
| 25-VB | O | NT | O | NT | O | E | E | NT | E | E | E | O |
| 26-PE | O | O | O | O | O | O | O | O | LOH | LOH | O | E |
| 27-BA | O | NT | E | NT | O | O | E | O | O | E | O | O |
| 28-ZF | O | O | E | E | E | O | O | O | O | LOH | LOH | O |
| 29-FJ | E | O | E | O | E | E | E | E | E | E | NT | E |
| 30-TV | O | O | O | O | O | E | E | E | E | E | E | O |
| 31-PG | O | O | O | O | O | O | NT | O | E | E | E | O |
| 32-PI | E | O | E | O | E | E | O | O | O | O | E | O |
| 33-ME | E | E | O | O | E | E | O | O | O | E | O | O |
| 34-CR | E | O | O | O | E | E | O | E | O | O | E | E |
| 35-GC | O | O | E | O | O | E | O | E | O | E | E | O |
| 36-DR | O | E | E | O | E | O | NT | O | O | O | E | E |
| 37-RD | O | LOH | LOH | O | O | LOH | LOH | O | O | O | LOH | LOH |
| 38-VC | O | E | O | O | O | E | O | E | O | E | E | O |
| 39-GL | LOH | LOH | LOH | O | O | O | O | LOH | O | LOH | NT | LOH |
| 40-PL | LOH | O | LOH | O | O | E | LOH | O | O | LOH | O | E |
| 41-RL | O | E | O | O | O | O | O | O | O | O | O | E |
| 42-MM | LOH | LOH | O | O | LOH | O | O | O | O | LOH | NT | LOH |
| 43-SP | LOH | O | LOH | LOH | LOH | O | O | O | LOH | O | LOH | O |
| 44-BG | O | O | O | O | LOH | LOH | O | O | LOH | LOH | LOH | O |

O, homozygous patients; E, heterozygous patients with no allelic loss in their tumour; LOH, heterozygous patients with loss of heterozygosity in their tumour; NT, not tested.

In fact 11 out of 18 deleted tumour samples (61%) were obtained from patients with liver and/or gallbladder metastasis; on the contrary only three out of the 26 non deleted tumours (11%) were derived from patients with an infiltrated liver and/or gallbladder (Table I). The difference between the two groups (deleted and non deleted tumours) was statistically significant ($\chi^2$ corrected = 9.872, $P = 0.002$). By considering only liver infiltration, 9/18 and 2/26 were observed to be positive. This difference was also significant ($\chi^2$ corrected = 8.023, $P = 0.005$).

### Discussion

Deletions on the short arm of chromosome 11 are considered as a common and important event in the development and/or progression of many cancers. Such allelic deletions, in fact, could involve loss of a tumour suppressor gene within the affected region of the chromosome (Marshall, 1991). Other studies reported 11p deletions in ovarian tumours with non-random frequencies of 30–50% (Ehlen & Dubeau, 1990; Lee *et al.*, 1990; Zheng *et al.*, 1991). In our previous study we found Ha-ras1 allelic loss in four out of seven informative cases and, Ha-ras1 mutations being absent, we could exclude a loss of competitive effect of mutated versus wild type *ras* allele (Viel *et al.*, 1991). In this much wider series of ovarian tumours several other genes located centromeric to Ha-ras1 locus on 11p were also analysed in order to determine the extension of the chromosome deletion.

The frequency of 11p LOH found in our ovarian carcinoma samples (at least 41%) is similar or even higher than those previously reported for Wilms and other tumours (Fearon *et al.*, 1985; Ludwig *et al.*, 1991; Mackay *et al.*, 1988; Reeve *et al.*, 1989) and is likely to reflect somatic events specifically involved in tumourigenesis. Deletion affecting chromosome 11p were not so frequent in the study of Sato *et al.* (24%), but the reason for this apparent discrepancy might be consequent upon the different number and type of the loci tested (Sato *et al.*, 1991). Furthermore our data indicate the existence of two hot spots of gene deletion on the short arm of chromosome 11, similarly to that described for Wilms tumours (Reeve *et al.*, 1989; Wadey *et al.*, 1990) and lung carcinomas (Weston *et al.*, 1989; Ludwig *et al.*, 1991).

The first hot spot of gene deletion in ovarian carcinomas, mapping on band 11p13 between D11S16 and Catalase loci, corresponds to the chromosome location of the WT-1 gene (Rose *et al.*, 1990). WT-1 gene encodes a putative transcription factor implicated in nephrogenesis and gonad development (Pelletier *et al.*, 1991*c*). Its involvement in urogenital development might explain the urogenital defects observed among hereditary cases of Wilms tumour patients. Moreover, deletions and/or sequence abnormalities of this gene have been described in Wilms tumours, those that have developed sporadically and those in individuals affected by WAGR

**Figure 2** Extents of allelic deletions in the ovarian tumours. In the upper part of the figure the 18 tumours showing 11p LOH are recorded; in the lower part of the figure the sequential tumour samples obtained from three patients are shown. Grey bars indicate regions where heterozygosity was retained; solid bars indicate deleted regions, whilst homozygous non informative and non tested sequences are represented by open bars. Position and extension of 11p15.5 **a**, and 11p13 **b**, hot spots of deletion are indicated on the top.

(Wilms tumour, Aniridia, Genitourinary malformation, mental Retardation) and Denys-Drash syndromes (Gessler *et al.*, 1990; Haber *et al.*, 1990; Pelleteir *et al.*, 1991*a*; Pelletier *et al.*, 1991*b*). At present, we cannot state that this growth regulatory gene is implicated in ovarian carcinogenesis. However, its restricted tissue-specific expression in adults (gonads, kidney, uterus, spleen and peritoneal layers between organs) and, in particular, the very high WT-1 expression levels observed in granulosa and epithelial cells of the normal ovary (Pelleteir *et al.*, 1991*c*), suggest that loss of a WT-1 allele may contribute to the aetiology of the 11p13 deleted ovarian tumours.

The second hot spot of gene deletion in ovarian carcinomas, mapping on band 11p15.5 and telomeric to the β Globin gene, corresponds to chromosome location of the putative WT-2, initially associated with the Beckwith Wiedemann syndrome (Koufos *et al.*, 1989). At present, there is no information about DNA sequences on 11p15.5 possibly involved in Wilms, ovarian and other tumours. According to other authors (Ehlen & Dubeau, 1990; Lee *et al.*, 1990; Zheng *et al.*, 1991) we found a remarkably high percentage of allelic losses (47%) at the Ha-ras1 locus. However, Ha-ras1 LOH is unlikely to imply a direct role of such oncogene in ovarian carcinogenesis since genetic alterations were never detected at the retained allele (van't Veer *et al.*, 1988; Viel *et al.*, 1991). More probably, Ha-ras1 LOH marks allelic loss of adjacent genomic sequences functionally involved in ovarian tumourigenesis. We are currently analysing, in ovarian carcinomas, the role of the gene encoding insulin like-growth factor II (IGF2), which maps close to Ha-ras1 and whose expression is altered in Wilms, rhabdomyosarcomas and hepatoblastomas, malignancies also characterised by 11p15.5 LOH (Scott *et al.*, 1985).

We do not know if the two putative tumour suppressor genes have similar functions in ovarian carcinogenesis. However, their independent deletion in some tumours of our series, as well as in Wilms tumours (Wadey *et al.*, 1990), suggests that they can contribute independently to the step-wise development of the fully malignant phenotype. Moreover, the concomitant deletion of both genomic regions in some tumours (Henry *et al.*, 1989), such as tumours 8-RR, 13-ZE and 40-PL with two separated areas of deletion, indicates that the two genes may act synergistically. At present, our analysis does not allow us to establish if the two 11p deletions in 8-RR, 13-ZE and 40-PL, but also in the other ovarian tumours with deletions extending apparently uninterrupted from 11p13 to 11p15.5, occurred on one chromosome or were derived from alternate deletions involving both paternal and maternal chromosomes. In any case, the high frequency of the concomitant 11p13 and 11p15.5 deletions suggests the importance of this event in ovarian carcinogenesis.

Correlations with clinicopathological parameters demonstrated that in the group of patients with the loss of 11p alleles in their tumour (18 cases), 61% had a diagnosis of liver and/or gallbladder metastasis, whereas this infiltration was present in only 11% of the patients with 11p non deleted tumours. The statistically significant association we observed between liver metastasis and loss of 11p polymorphic loci, leads us to hypothesise that in ovarian cancer the presumed

11p oncosuppressor genes are involved in tumour progression rather than initiation. The biological significance of this correlation could be interpreted with the acquisition of a particular metastatic phenotype (ability to colonise the liver) by the tumour cells which lose 11p genes. According to this hypothesis patient RA, free of metastatic liver invasion, did not display such allelic losses in none of the two peritoneal tumour samples (16-RA and 17-RA); patient RR displayed 11p LOH late in the tumour evolution (tumour 8-RR), when a liver metastasis was diagnosed; finally patient ZE developed 11p LOH in her primary tumour (13-ZE) before clinical diagnosis of hepatic metastasis. These observations bring into question the possibility of a predictive role of 11p LOH analysis in the identification of patients at high risk to develop liver metastasis. More extensive studies, however, are required to reach a firm conclusion on this point and we are planning to extend this study to a larger number of new selected cases to define the reliability of this molecular marker.

The authors thank Dr G. Saglio for providing the β and γGlobin probes; Dr J.W.M. Höppener for the Calcitonin probe; Dr D.E. Housmann for D11S17 probe; Mr R. Harvey and Integrated Genetics, Inc (Framingham, Massachussetts) for the β-FSH probe. They are grateful to Miss Paola Pistello for her expert secretarial assistance.

### References

ALI, I.U., LIDERAU, R., THEILLET, C. & CALLAHAM, R. (1987). Reduction to homozygosity of genes on chromosome 11 in human breast neoplasia. *Science*, **238**, 185-188.

BARKER, D., HOLM, T. & WHITE, R. (1984). A locus on chromosome 11p with multiple restriction site polymorphisms. *Am. J. Hum. Genet.*, **36**, 1159-1171.

BELL, G.I., KARAM, J.H. & RUTTER, W.J. (1981). Polymorphic DNA region adjacent to the 5' end of the human insulin gene. *Proc. Natl Acad. Sci. USA*, **78**, 5759-5763.

BOLTZ, E.M., KEFFORD, R.F., LEARY, J.A., HOUGHTON, C.R. & FRIEDLANDER, M.L. (1989). Amplication of c-ras-Ki oncogene in human ovarian tumors. *Int. J. Cancer*, **43**, 428-430.

DEVILEE, P., VAN DEN BROEK, M., MANNENS, M., SLATER, R., CORNELISSE, C.J., WESTERVELD, A. & KHAN, M. (1991). Differences in patterns of allelic loss between two common types of adult cancer, breast and colon carcinoma, and Wilms' tumor of childhood. *Int. J. Cancer*, **47**, 817-821.

ECCLES, D.M., CRANSTON, G., STEEL, C.M., NAKAMURA, Y. & LEONARD, R.C.F. (1990). Allele losses on chromosome 17 in human epithelial ovarian carcinoma. *Ocogene*, **5**, 1599-1601.

EHLEN, T. & DUBEAU, L. (1990). Loss of heterozygosity on chromosomal segments 3p, 6q and 11p in human ovarian carcinomas. *Oncogene*, **5**, 219-223.

ENOMOTO, T., WEGHORST, C.M., INOUE, M., TAIZAWA, O. & RICE, M. (1991). Ki-ras activation occurs frequently in mucinous adenocarcinomas and rarely in other common epithelial tumors of the human ovary. *Am. J. Pathol.*, **139**, 777-785.

FEARON, E.R., FEINBERG, A.P., HAMILTON, S.H. & VOGELSTEIN, B. (1985). Loss of genes on the short arm of chromosome 11 in bladder cancer. *Nature*, **318**, 377-380.

FEDER, J., YEN, L., WIJSMAN, E., WANG, L., WILKINS, L., SCHRODER, J., SPURR, N., CANN, H., BLUMENBERG, M. & CAVALLI-SFORZA, L.L. (1985). A systematic approach for detecting high frequency restriction fragment length polymorphisms using large genomic probes. *Am. J. Hum. Genet.*, **37**, 635-649.

GESSLER, M., POUSTKA, A., CAVANEE, W., NEVE, R.L., ORKIN, S.H. & BRUNS, G.A.P. (1990). Homozygous deletion in Wilms tumours of a zinc-finger gene identified by chromosome jumping. *Nature*, **343**, 774-778.

HABER, D.A., BUCKLER, A.J., GLASER, T., CALL, K.M., PELLETIER, J., SOHN, R.L., DOUGLASS, E.C. & HOUSMAN, D.E. (1990). An internal deletion within an 11p13 zinc finger gene contributes to the development of Wilms' tumor. *Cell*, **61**, 1257-1269.

HENRY, I., GRANDJOUAN, S., COUILLIN, P., BARICHARD, F., HUERRE-JEANPIERRE, C., GLASER, T., PHILIP, T., LENOIR, G., CHAUSSAIN, J.L. & JUNIEN, C. (1989). Tumor-specific loss of 11p15.5 alleles in del11p13 Wilms tumor and in familial adrenocortical carcinoma. *Proc. Natl. Acad. Sci.*, **86**, 3247-3251.

HÖPPENER, J.W.M., STEENBERGH, P.H., ZANDBERG, J., BAKKER, E., PEARSON, P.L., GEURTS VAN KESSEL, A.H.M., JANSZ, H.S. & LIPS, C.J.M. (1984). Localization of the polymorphic human calcitonin gene on chromosome 11. *Hum. Genet.*, **66**, 309-312.

HOUSMAN, D.E., GERHARD, D.S., GLASER, T. & JONES, C. (1985). Mapping of chromosome 11p by linkage and somatic cell genetic technique: A comparison of relative map order derived by each method. Human Gene Mapping VIII. *Cytogenet. Cell Genet.*, **40**, 656.

KOUFOS, A., HANSEN. M.F., COPELAND, N.G., JENKINS, N.A., LAMPKIN, B.C. & CAVANEE, W.K. (1985). Loss of heterozygosity in three embryonal tumours suggests a common pathogenetic mechanism. *Nature*, **316**, 330-334.

KOUFOS, A., GRUNDY, P., MORGAN, K., ALECK, K.A., HADRO, T., LAMPKIN, B.C., KALBAKJI, A. & CAVANEE, W.K. (1989). Familial Wiedemann-Beckwith Syndrome and a second Wilms tumor locus both map to 11p15.5. *Am. J. Hum. Genet.*, **44**, 711-719.

LEE, J.H., KAVANAGH, J.J., WILDRICK, D.M., WHARTON, J.T. & BLICK, M. (1990). Frequent loss of heterozygosity on chromosomes 6q, 11, and 17 in human ovarian carcinomas. *Cancer Res.*, **50**, 2724-2728.

LI, S., SCHWARTZ, P.E., LEE, W. & YANG-FENG, T.L. (1991). Allele loss at the retinoblastoma locus in human ovarian cancer. *J. Natl Cancer Inst.*, **83**, 637-640.

LOTHE, R.A., FOSSA, S.D., STENWIG, A.E. & 4 others (1989). Loss of 3p or 11p alleles is associated with testicular cancer tumors. *Genomics*, **5**, 134-138.

LUDWIG, C.U., RAEFLE, G., DALQUEN, P., STULZ, P., STAHEL, R. & OBRECHT, J. (1991). Allelic loss on the short arm of chromosome 11 in non-small-cell lung cancer. *Int. J. Cancer*, **49**, 661-665.

MACKAY, J., ELDER, P.A., PORTEOUS, D.J., STEEL, C.M., HAWKINS, R.A., GOING, J.J. & CHETTY, U. (1988). Partial deletion of chromosome 11p in breast cancer correlates with size of primary tumour and oestrogen receptor level. *Br. J. Cancer*, **58**, 710-714.

MARSHALL, C.J. (1991). Tumor suppressor genes. *Cell*, **64**, 313-326.

OKAMOTO, A., SAMESHIMA, Y., YOKOYAMA, S., TERASHIMA, Y., SUGIMURA, T., TERADA, M. & YOKOTA, J. (1991). Frequent allelic losses and mutations of the p53 gene in human ovarian cancer. *Cancer Res.*, **51**, 5171-5176.

PELLETIER, J., BRUENING, W., KASHTAN, C.E., MAUER, S.M., MANIVEL, J.C., STRIEGEL, J.E., HOUGHTON, D.C., JUNIEN, C., HABIB, R., FOUSER, L., FINE, R.N., SILVERMAN, B.L., HABER, D.A. & HOUSMAN, D. (1991*a*). Germline mutations in the Wilms' tumor suppressor gene are associated with abnormal urogenital development in Denys-Drash Syndrome. *Cell*, **67**, 437-447.

PELLETIER, J., BRUENING, W., LI, F.P., HABER, D.A., GLASER, T. & HOUSMAN, D.E. (1991*b*). WT1 mutations contribute to abnormal genital system development and hereditary Wilms' tumour. *Nature*, **353**, 431-434.

PELLETIER, J., SCHALLING, M., BUCKLER, A.J., ROGERS, A., HABER, D.A. & HOUSMAN, D. (1991*c*). Expression of the Wilms' tumor gene WT1 in the murine urogenital system. *Genes & Dev.*, **5**, 1345-1356.

PULCIANI, S., SANTOS, E., LAUVER, A.V., LONG, L.K. & BARBACID, M. (1982). Transforming genes in human tumors. *J. Cell. Biochem.*, **20**, 51-61.

QUAN, F., KORNELUCK, R.G., MACLEOD, H.L., TSUI, L.C. & GRAVEL, R.A. (1985). An RFLP associated with the human catalase gene. *Nucl. Acid Res.*, **13**, 8288.

REEVE, A.E., SIH, S.A., RAIZIS, A.M. & FEINBERG, A.P. (1989). Loss of allelic heterozygosity at a second locus on chromosome 11 in sporadic Wilms' tumor cells. *Mol. Cell. Biol.*, **9**, 1799-1803.

ROSE, E.A., GLASER, T., JONES, C., SMITH, C.L., LEWIS, W.H., CALL, K.M., MINDEN, M., CHAMPAGNE, E., BONETTA, L., YEGER, H. & HOUSMAN, D.E. (1990). Complete physical map of the WAGR region of 11p13 localizes a candidate Wilms' tumor gene. *Cell*, **60**, 495-508.

RUSSEL, S.E.H., HICKEY, G.I., LOWRY, W.S., WHITE, P. & ATKINSON, R.J. (1990). Allele loss from chromosome 17 in ovarian cancer. *Oncogene*, **5**, 1581-1583.

SAMBROOK, J., FRITSCH, E.F. & MANIATIS, T. (1989). Appendix E: Commonly used techniques in molecular cloning. In *Molecular Cloning: a Laboratory Manual.* Nolan, C. (ed), Colg Spring Harbor: New York.

SATO, T., SAITO, H., MORITA, R., KOI, S., LEE, J.H. & NAKAMURA, Y. (1991). Allelotype of human ovarian cancer. *Cancer Res.*, **51**, 5118-5122.

SCOTT, J., COWELL, J., ROBERTSON, M.E., PRIESTLEY, L.M., WADEY, R., HOPKINS, B., PRITCHARD, J., BELL, G.I., RALL, L.B., GRAHAM, C.F. & KNOTT, T.J. (1985). Insulin-like growth factor II gene expression in Wilms' tumour and embryonic tissues. *Nature*, **317**, 260-262.

SCRABLE, H.J., WITTE, D.P., LAMPKIN, B.C. & CAVANEE, W.K. (1987). Chromosomal localization of the human rhabdomyosarcoma locus by mitotic recombination mapping. *Nature*, **329**, 645–647.

SLAMON, D.J., GODOLPHIN, W., JONES, L.A., HOLT, J.A., WONG, S.G., KEITH, D.E., LEVIN, W.J., STUART, S.G., UDOVE, J., ULLRICH, A. & PRESS, M.F. (1989). Studies of the HER-2/neu proto-oncogene in human breast and ovarian cancer. *Science*, **244**, 707–712.

VAN'T VEER, L.J., HERMENS, R., VAN DEN BERG-BAKKER, L.A.M., CHENG, N.C., FLEUREN, G-J., BOS, J.L., CLETON, F.J. & SCHRIER, P.I. (1988). ras oncogene activation in human ovarian carcincoma. *Oncogene*, **2**, 157–165.

VIEL, A., MAESTRO, R., TOFFOLI, G., GRION, G. & BOIOCCHI, M. (1990). c-*myc* overexpression is a tumor-specific phenomenon in a subset of human colorectal carcinomas. *J. Cancer Res. Clin. Oncol.*, **116**, 288–294.

VIEL, A., DE PASCALE L., TOFFOLI, G., TUMIOTTO, L., MIOTTO, E. & BOIOCCHI, M. (1991). Frequent occurrence of Ha-ras1 allelic deletion in human ovarian adenocarcinomas. *Tumori*, **77**, 16–20.

WADEY, R.B., PAL, N., BUCKLE, B., YEOMANS, E., PRITCHARD, J. & COWELL, J.K. (1990). Loss of heterozygosity in Wilms' tumour involves two distinct regions of chromosome 11. *Oncogene*, **5**, 901–907.

WATKINS, P.C., EDDY, A., BECK, A.K., VELLUCCI, V., LEVERONE, B., TANZI, R.E., GUSELLA, J.F. & SHOWS, T.B. (1987) DNA sequence and regional assignment of the human follicle-stimulating hormone $\beta$-subunit gene to the short arm of chromosome 11. *DNA*, **6**, 205–212.

WESTON, A., WILLEY, J.C., MODALI, R., SUGIMURA, H., MCDOWELL, E.M., RESAU, J., LIGHT, B., HAUGEN, A., MANN, D.L., TRUMP, B.F. & HARRIS, C.C. (1989). Differential DNA sequence deletions from chromosomes 3, 11, 13 and 17 in squamous-cell carcinoma, large-cell carcinoma, and adenocarcinoma of the lung. *Proc. Natl Acad. Sci.*, **86**, 5099–5103.

XIANG, K., KARAM, J.H. & BELL, G.I. (1987). BamHI RFLP at the insulin-like growth factor II (IGF2) locus on chromosome 11. *Nucl. Acid Res.*, **15**, 7655.

ZHENG, J., ROBINSON, W.R., EHLEN, T., YU, M.C. & DUBEAU, L. (1991). Distinction of low grade from high grade human ovarian carcinomas on the basis of losses of heterozygosity on chromosomes 3, 6, and 11 and HER-2/neu gene amplification. *Cancer Res.*, **51**, 4045–4051.

ZHOU, D.J., GONZALES-CADAVID, N., AHUJA, H., BATTIFORA, H., MOORE, G.E. & CLINE, M.J. (1988). A unique pattern of protoocogene abnormalities in ovarian adenocarcinomas. *Cancer*, **62**, 1573–1576.